# Supplementary material for: Commissioning an All-Sky Infrared Camera Array for Detection of Airborne Objects
Source: Sensors (Basel). 2025 Jan 28;25(3):783. doi: 10.3390/s25030783 (PMC11820869; doi:10.3390/s25030783)
Supplement: Supplementary file 1 [file sensors-25-00783-s001.zip › sensors-3357257-supplementary.pdf]

Supplementary Materials: Commissioning An All-Sky Infrared Camera Array for Detection Of Airborne Objects

Laura Domine [1,3,*], Ankit Biswas [3], Richard Cloete [1,3], Alex Delacroix [1,3], Andriy Fedorenko [3], Lucas Jacaruso [3], Ezra Kelderman [3], Eric Keto [1,3], Sarah Little [2,3,4], Abraham Loeb [1,3,†], Eric Masson [3], Mike Prior [3], Forrest Schultz [3,5], Matthew Szenher [3], Wesley Andrés Watters [2,3], Abigail White [1,3]

1. Examples of manually identified trajectories

We hand picked some representative trajectories from our commissioning dataset that we manually identified as birds, leaves, or clouds.

1.1. Birds

Figure S1 shows trajectories with IDs 39 and 306 that include multiple loops (sinuosity > 3). The object exhibits an unusually long trajectory lifespan. The trajectory with ID 306 re-entered the scene and exhibited a similar behavior, generating additional looping trajectories such as those labeled 322, 320, and 300. Visual inspection identified these objects as birds, presumably hawks or eagles that loop around thermals and may hover for a short period of time. The next Figure S2 presents another looping trajectory (Figure S2C and Figure S2D). The same figure also shows an interesting object with sinuosity < 3 in Figure S2A and Figure S2B: the trajectory with ID 14 starts in the upper right corner (ID 12) and then moves sharply downward, decelerating as it transitions into a straight trajectory labeled ID 14. This motion suggests a change in velocity, marked by an initial curved path followed by a more linear motion pattern.

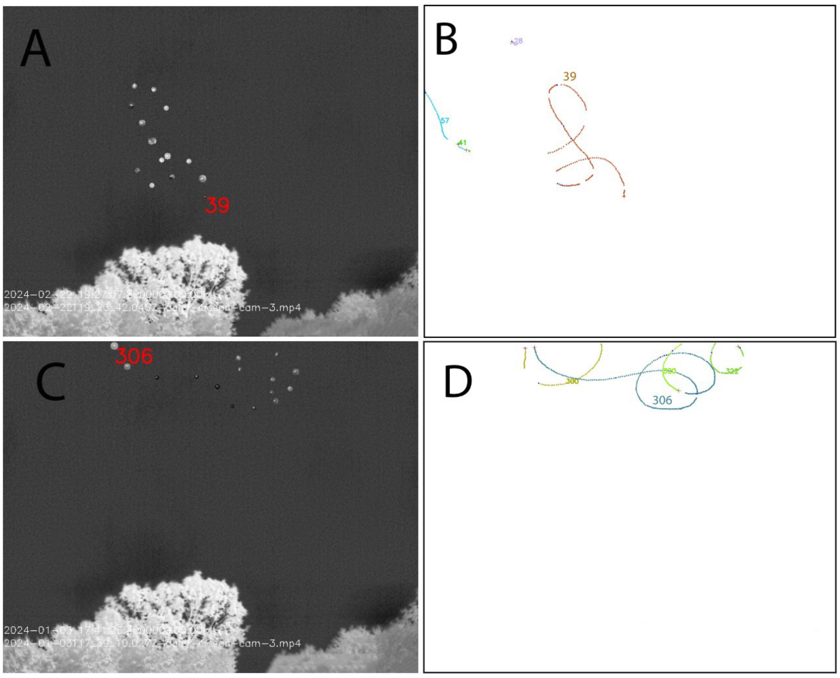

Figure S1. Left: Camera background image, overlaid with snapshots from the objects' detections along their trajectories for two specific trajectories with IDs 39 and 306, identified as hawks after manual examination. Right: All reconstructed trajectories in a given day, including trajectories 39 and 306, summarized by assigning a unique color and identifier to each point of these trajectories.

Figure S3 presents another example of a looping trajectory. The object makes a sharp U-turn and moves backward. We also identify objects traveling in pairs or as part of a larger formation, changing their alignment as they move. These are identified as flocks of birds, as illustrated in Figure S4.

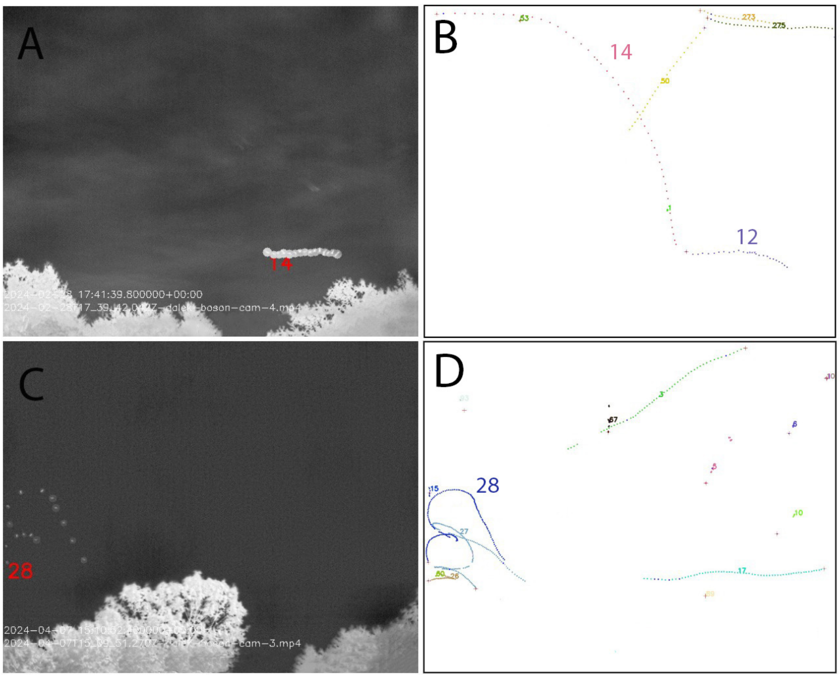

Figure S2. Left: Camera background image, overlaid with snapshots from the objects' detections along their trajectories for two specific trajectories with IDs 14 and 28, identified as hawks after manual examination. Right: All reconstructed trajectories in a given day, including trajectories 14 and 28, summarized by assigning a unique color and identifier to each point of these trajectories.

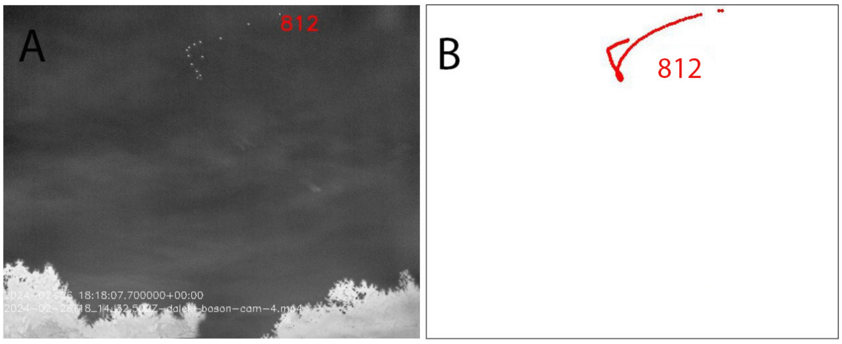

Figure S3. Left: Camera background image, overlaid with snapshots from the object's detections along its trajectory, for the specific trajectory with ID 812, identified as a hawk after manual examination. Right: All reconstructed trajectories in a given day, including trajectory 812, summarized by assigning a unique color and identifier to each point in these trajectories.

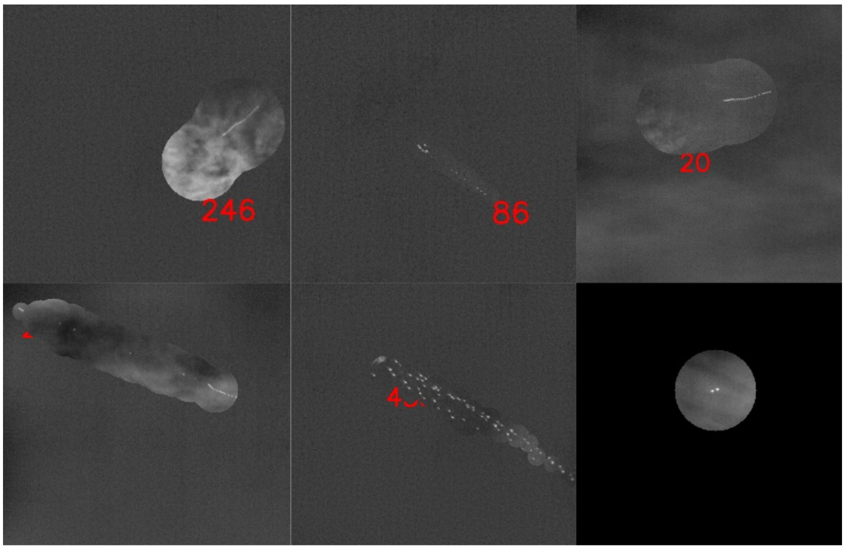

Figure S4. Zoomed view of the individual object detections overlaid along different reconstructed trajectories. The red number is the unique identifier assigned to each trajectory by the tracking algorithm (SORT). All of these examples were identified as flocks of birds after manual examination.

1.2. Leaves

We also find interesting trajectories where the objects change their shape from frame to frame, such as in Figure S5. Visual inspection of the recordings helps to identify trajectories 37, 115, and 18 as leaves flying off and toward the camera.

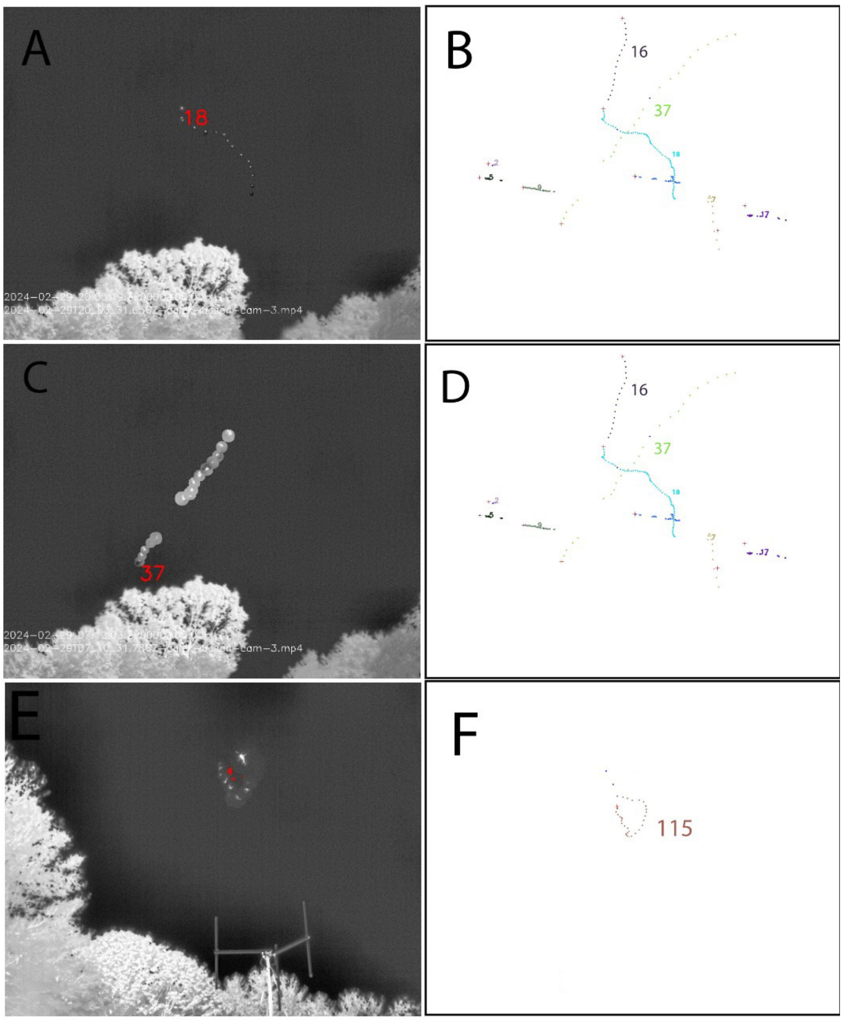

Figure S5. Left: Camera background image, overlaid with snapshots from the objects' detections along their trajectories for three specific trajectories with IDs 18, 37, and 115, which were all identified after manual examination as leaves. Right: All reconstructed trajectories in a given day, including trajectories 18, 37, and 115, summarized by assigning a unique color and identifier to each point of these trajectories.

1.3. Clouds

The next set of objects are clouds of different shapes. Manual labeling shows that 35% of our trajectories are made by clouds, which are shown in Figure S6.

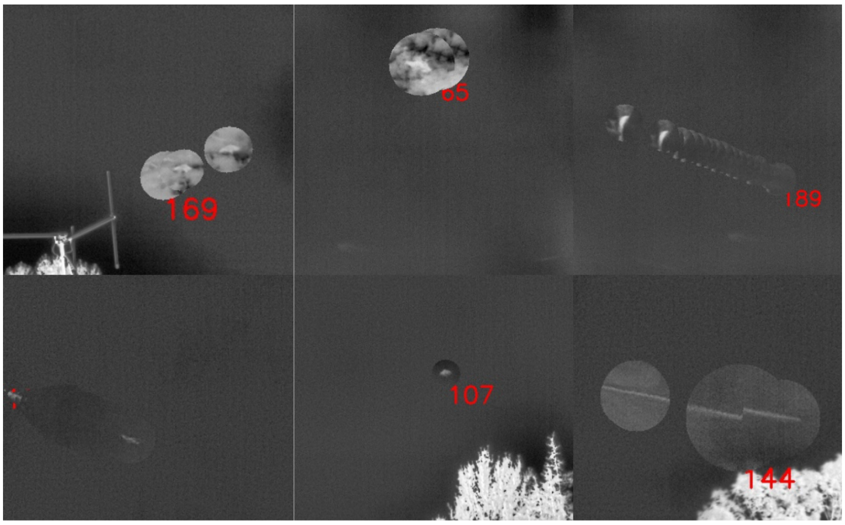

Figure S6. Zoom in on the individual object detections overlaid along different reconstructed trajectories. The red number is the unique identifier assigned to each trajectory by the tracking algorithm (SORT). All of these examples were identified as clouds after manual examination.

2. Order-of-magnitude range estimation using common known object speeds

All-sky cameras alone face several limitations due to the lack of range data, which affects our ability to accurately interpret and detect objects and their trajectories. The scale ambiguity makes it difficult to differentiate between nearby and distant objects (such as those in Figure S4), leading to challenges in estimating their size, speed, and altitude. Distant objects may appear blurred, and atmospheric distortions further degrade image clarity, making detection harder. Additionally, without range information, it is a challenge to accurately track object speed or compensate for motion blur, especially for apparently fast-moving objects like bugs or birds flying very close to the camera. For example, in Figure S5B, the trajectories with IDs 18, 37, and 15 show disrupted, looping shapes. In this case, visual inspection resolves the nature of the objects (leaves), but even this simple approach fails when we need to differentiate objects measuring only a few pixels across.However, given an observed angular speed and an estimate of the most common average speed of given objects classes, we can approximately estimate the order of magnitude for the range of the object from the camera. For example, in Figure S5A, C, and E, leaves cross a 50-degree field of view in 15 seconds, 5 seconds, and 3 seconds, respectively, with wind-driven speeds typically ranging from 2–5 m/s. By combining the observed crossing times and speeds, we estimate the approximate order of magnitude for distances from the camera to the leaves, which are 5, 11, and 35 meters, respectively. Similarly, taking the average speed of birds (e.g., hawks when flying) as 10–16 m/s, the time needed to cross the field of view in Figure S1A and C, which is 10 and 18 seconds, gives distances of approximately 150 and 270 meters, respectively. In Figure S4, flocks of birds corresponding to the trajectories with IDs 246, 86, and 20, are estimated to be at a range of about 1.3 km, crossing the field of view within 42 seconds with the speed of a flock of geese is estimated to be about 60 mph (25 m/s). To provide a more accurate estimate of the distance to the object, range estimation techniques such as optical triangulation [1] or passive radar [2] would be required. Both are in development for our observatory.

References

1. Szenher, M.; Delacroix, A.; Keto, E.; Little, S.; Randall, M.; Watters, W.A.; Masson, E.; Cloete, R. A hardware and software platform for aerial object localization. *Journal of Astronomical Instrumentation* **2023**, *12*, 2340002.
2. Randall, M.; Delacroix, A.; Ezell, C.; Kelderman, E.; Little, S.; Loeb, A.; Masson, E.; Watters, W.A.; Cloete, R.; White, A. Skywatch: A passive multistatic radar network for the measurement of object position and velocity. *Journal of Astronomical Instrumentation* **2023**, *12*, 2340004.

Disclaimer/Publisher's Note: The statements, opinions and data contained in all publications are solely those of the individual author(s) and contributor(s) and not of MDPI and/or the editor(s). MDPI and/or the editor(s) disclaim responsibility for any injury to people or property resulting from any ideas, methods, instructions or products referred to in the content.
